# Supplementary material for: Immune Analysis Using Vitreous Optical Coherence Tomography Imaging in Rats with Steroid-Induced Glaucoma
Source: Biomedicines. 2024 Mar 13;12(3):633. doi: 10.3390/biomedicines12030633 (PMC10967960; doi:10.3390/biomedicines12030633)
Supplement: Supplementary file 1 [file biomedicines-12-00633-s001.zip › biomedicines-2881588-supplementary.pdf]

SUPPLEMENTARY FIGURE S1.

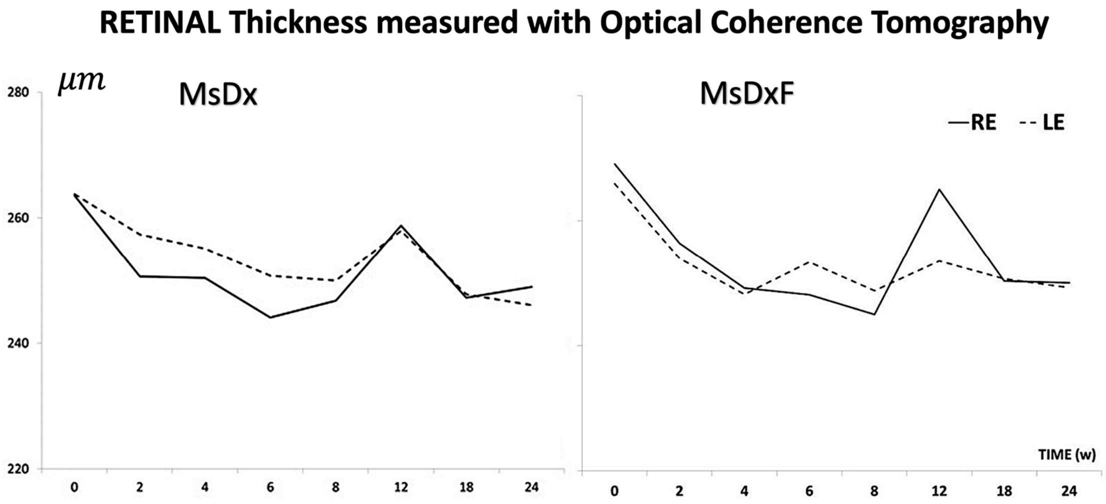

Supplementary Figure S1: Retinal thickness measured with OCT. Abbreviations: MsDx: cohort with microspheres loaded with dexamethasone; MsDxF: cohort with microspheres loaded with dexamethasone and fibronectin injected into the anterior chamber, RE: right eye; LE: left eye; w: weeks. Data extracted from: [19,20] (CYB license).
